# Supplementary material for: Genetic and environmental interactions contribute to immune variation in rewilded mice
Source: Nat Immunol. 2024 Jun 14;25(7):1270–82. doi: 10.1038/s41590-024-01862-5 (PMC11224019; doi:10.1038/s41590-024-01862-5)
Supplement: Supplementary file 1 — Reporting Summary [file 41590_2024_1862_MOESM1_ESM.pdf]

nature portfolio

Corresponding author(s): Oyebola Oyesola, Andrea Graham, P'ng Loke

Last updated by author(s): Apr 29, 2024

Reporting Summary

Nature Portfolio wishes to improve the reproducibility of the work that we publish. This form provides structure for consistency and transparency in reporting. For further information on Nature Portfolio policies, see our Editorial Policies and the Editorial Policy Checklist.

Statistics

For all statistical analyses, confirm that the following items are present in the figure legend, table legend, main text, or Methods section.

n/a	Confirmed	
☐	☒	The exact sample size (*n*) for each experimental group/condition, given as a discrete number and unit of measurement
☐	☒	A statement on whether measurements were taken from distinct samples or whether the same sample was measured repeatedly
☐	☒	The statistical test(s) used AND whether they are one- or two-sided<br>*Only common tests should be described solely by name; describe more complex techniques in the Methods section.*
☐	☒	A description of all covariates tested
☐	☒	A description of any assumptions or corrections, such as tests of normality and adjustment for multiple comparisons
☐	☒	A full description of the statistical parameters including central tendency (e.g. means) or other basic estimates (e.g. regression coefficient) AND variation (e.g. standard deviation) or associated estimates of uncertainty (e.g. confidence intervals)
☐	☒	For null hypothesis testing, the test statistic (e.g. *F*, *t*, *r*) with confidence intervals, effect sizes, degrees of freedom and *P* value noted<br>*Give P values as exact values whenever suitable.*
☐	☒	For Bayesian analysis, information on the choice of priors and Markov chain Monte Carlo settings
☐	☒	For hierarchical and complex designs, identification of the appropriate level for tests and full reporting of outcomes
☐	☒	Estimates of effect sizes (e.g. Cohen's *d*, Pearson's *r*), indicating how they were calculated

Our web collection on statistics for biologists contains articles on many of the points above.

Software and code

Policy information about availability of computer code

Data collection

Blood samples (approx. 30-50�L) were collected from all mice at endpoint via cheek bleeds using a Medipoint Golden Rod Lancet Blade 4MM (Medipoint NC9922361) into a 1.3mL heparin coated tube (Sarstedt Inc, NC9574345). Blood samples were analyzed using the element HT5, Veterinary Hematology Analyzer (Heska).

All Flow Cytometry samples were collected on an Aurora™ spectral cytometer (Cytek) and analyzed using the OMIQ software (https://www.omiq.ai/), data cleaning and scaling was done using algorithms like FlowCut56, 57 within the OMIQ software

Cytokine concentrations in plasma or MLN supernatants were measured also measured using the commercially available murine Th cytokine LEGENDplex assay (Biolegend) panel (Cat #741044) according to the manufacturer's instructions.

Single cell suspensions were obtained from MLN and pooled samples from each group were then loaded on a 10X Genomics platform, processed to generate cDNA and sequencing libraries were performed following 10X Genomics' protocol. Libraries were pooled and sequenced using Illumina NovaSeq SP 100 cycle as per 10X sequencing recommendations.

Data analysis

In all cases principal component analysis was performed with in R v4.1.2. For cytokine data, log transformed data were used for generation of PCA plots and for MDMR analysis. Biplots were constructed by projecting the weighted averages of each input feature (immune cell phenotypes, cytokine level, cellular composition etc.,) along PC1, PC2 and/or PC3 derived from the biplot.pcoa function from the ape package

The sequenced data were processed using Cell Ranger (version 6.0) to demultiplex the libraries. The reads were aligned to Mus musculus mm10 genomes to generate count tables that were further analyzed using Seurat (version 4.0). Sequencing data from the two blocks were

integrated together prior to further downstream analysis.

Statistical analysis was performed using GraphPad Prism software (v9). Right-skewed data were log or square root transformed. For analysis of relationship between scRNAseq cell composition and worm burden in Fig. 5, worm burden was modeled as following a negative binomial distribution. Predictor variables included in the regression model were mouse strain, mouse environment (i.e., laboratory or rewilded), and loading on PC2 from analysis of scRNAseq data.

Code used for the analysis can be found here - https://github.com/oyeb2003/G-ERewilded-interaction-Project.

For manuscripts utilizing custom algorithms or software that are central to the research but not yet described in published literature, software must be made available to editors and reviewers. We strongly encourage code deposition in a community repository (e.g. GitHub). See the Nature Portfolio guidelines for submitting code & software for further information.

Data

Policy information about availability of data

All manuscripts must include a data availability statement. This statement should provide the following information, where applicable:

- Accession codes, unique identifiers, or web links for publicly available datasets
- A description of any restrictions on data availability
- For clinical datasets or third party data, please ensure that the statement adheres to our policy

Raw scRNA sequencing data are deposited to the NCBI Sequence Archive (GSE236347). All other data needed to support the conclusions of the paper are present in the paper, Data Files or Extended Data Figures or can be accessed here - doi:10.5061/dryad.ncjsxkt3w. If you need any more details about any of the data set, please contact lead corresponding author of the paper – Dr. P'ng Loke. - png.loke@nih.gov

Research involving human participants, their data, or biological material

Policy information about studies with human participants or human data. See also policy information about sex, gender (identity/presentation), and sexual orientation and race, ethnicity and racism.

Reporting on sex and gender	N/A
Reporting on race, ethnicity, or other socially relevant groupings	N/A
Population characteristics	N/A
Recruitment	N/A
Ethics oversight	N/A

Note that full information on the approval of the study protocol must also be provided in the manuscript.

Field-specific reporting

Please select the one below that is the best fit for your research. If you are not sure, read the appropriate sections before making your selection.

☒ Life sciences ☐ Behavioural & social sciences ☐ Ecological, evolutionary & environmental sciences

For a reference copy of the document with all sections, see nature.com/documents/nr-reporting-summary-flat.pdf

Life sciences study design

All studies must disclose on these points even when the disclosure is negative.

Sample size	25- 30 mice of mixed strains and genotype, 129S1/SV1mJ, C57BL/6J and PWK/PhJ female mice were used for these experiments. Sample size was determined by logistical constraints and not by power calculations. For rewilding, 12-18 female mice of the different strains (129S1/SV1mJ, C57BL/6J and PWK/PhJ) were housed in different wedges in the enclosure for 5 weeks. In summary, for Block 1 we rewilded, n = 42 mice (15 PWK/PhJ, 14 C57BL/6, 13 129S1); in Block 2 we rewilded, n = 47 mice (16 PWK/PhJ, 18 C57BL/6, 13 129S1) for a total of n =89 rewilded mice
Data exclusions	For all analysis, samples that failed pre-determined quality control such as flow cytometry staining errors, high cell death and/or are under limit of detection such as for the ELISA assay are not included in downstream statistical analyses. For the different measurements and assays, the same sample size was measured repeatedly except were mentioned in the Figure Legends. The number of mice per group, number of experimental replicates if any, and the statistical tests employed are reported in the figure legends. All data points represent biological replicates.
Replication	Experiments were repeated across two different experiment blocks with some immune phenotypes that replicates previously published rewilding studies. All data points represent biological replicates except where mentioned in the figure legend

Randomization	Mice were randomly assigned to either remain in the institutional vivarium (lab mice) or released into the outdoor enclosures (rewilded mice) previously described. For all experiments, only female mice were used to prevent unintended breeding in the rewilded environment and mice were between 8-12 weeks at point of blood draw following rewilding
Blinding	Investigators were blinded to the experimental groups the mice belong to at the time of the performing the different experimental assay but were unblinded at the point of statistical analysis and testing.

Reporting for specific materials, systems and methods

We require information from authors about some types of materials, experimental systems and methods used in many studies. Here, indicate whether each material, system or method listed is relevant to your study. If you are not sure if a list item applies to your research, read the appropriate section before selecting a response.

Materials & experimental systems

n/a	Involved in the study
☐	☒ Antibodies
☒	☐ Eukaryotic cell lines
☒	☐ Palaeontology and archaeology
☐	☒ Animals and other organisms
☒	☐ Clinical data
☒	☐ Dual use research of concern
☒	☐ Plants

Methods

n/a	Involved in the study
☒	☐ ChIP-seq
☐	☒ Flow cytometry
☒	☐ MRI-based neuroimaging

Antibodies

Antibodies used

Antibodies were used for the following antigens (clone) at the stated concentration from the stated vendor (catalog number)

Lymphoid Panel
Anti-mouse CD45 (30-F11) BUV395 BD Biosciences Cat#: 564279; 1:300
Rat Anti-mouse CD103 (M290) BUV496 BD Biosciences Cat#: 741083; 1:200
Rat Anti-mouse CD62L (MEL-14) BUV563 BD Biosciences Cat#: 741230; 1::100
Hamster Anti-mouse TCR-B chain (H57-597) BUV737 BD Biosciences Cat#: 612821; 1:200
Rat Anti-Mouse CD44 (IM7) BUV805 BD Biosciences Cat#: 741921; 1:100
Rat Anti-Mouse NKp46 CD335 (29A1.4), BV480 BD Biosciences Cat#: 746264 1:50
Anti-Mouse NK-1.1 (PK136), BUV661 BD Biosciences Cat#:74147; 1:200
Anti-mouse CD8β (H35-17.2) BV510 BD Biosciences Cat#: 740155; 1:200
Anti-mouse CD4 (RM4-5) BV570 BioLegend Cat#: 100542: 1:200
Hamster Anti-Mouse KLRG1 (2F1), BV750 BD Biosciences Cat#: 746972; 1:100
Anti-mouse TCRγδ (eBio-GL3) PECy5 Thermofisher Cat#: 15-5711-82 ; 1:50
Anti-mouse CD45R/B220 (RA3-6B2) APC/Fire810 BioLegend Cat#: 103278 1:200
Ant-mouse Tbet (4B10) BV421 Biolegend Cat#: 644816 1:50
Ki-67 Monoclonal Antibody (SolA15) AF532 Thermofisher Cat#: 58-5698-82; 1:100
Anti-mouse FoxP3 (FJK-16) AF700 Thermofisher Cat#: 56-5773-82 1:100
Anti-mouse RorgT (Q31-378) PE-CF594 BD Biosciences Cat#: 56284 1:50
Gata-3 Monoclonal Antibody (TWAJ), Alexa Fluor™ 488 Thermofisher Cat#: 53-9966-42; 1:50
Hamster Anti-Mouse CD69 (H1.2F3) BV650 BD Biosciences Cat#: 740460; 1:200
Anti-Mouse CD279 (PD-1) (29F.1A12) BV421 BioLegend Cat#:135218; 1:100
Anti-mouse IL-4 Clone 11B11 Alexa Flour 488 BD Biosciences Cat#: 557728; 1:50
Anti-mouse IFN-γ Antibody Clone XMG1.2 PerCP/Cy5.5 Biolegend Cat#: 505822; 1:100
Anti-mouse IL-13 Antibody Clone (eBio13A) PE-Cyanine 7 Thermofisher Cat#: 25-7133-82; 1:50

For Myeloid Panel.
Anti-mouse CD45 (30-F11) BUV395 BD Biosciences Cat#: 564279; 1:300
Rat Anti-mouse CD43 (S7) BUV563 BD Biosciences Cat#: 741238;1:100
Anti-mouse CD11b (M1/70) BUV615 BD Biosciences Cat#: 7511401:200
Anti-mouse TCRβ (H57-597) BUV661 BD Biosciences Cat#: 749914 1:200
Anti-mouse CD44 (IM7) BUV805 BD Biosciences Cat#: 741921 1:200
Anti-Mouse CD279 (PD-1) (29F.1A12) BV421 BioLegend Cat#: 135218; 1:150
MERTK Monoclonal Antibody (DS5MMER), Super Bright 436 Thermofisher Cat#: 62-5751-82 1:50
Anti-mouse CD49b (DX5), Pacific Blue Biolegend Cat #: 108918; 1:100
Rat Anti-mouse F4/80 Anti-Mouse (T45-2342) F4/80 BD Bioscience Cat #: 565635 1:100
Hamster Anti-Mouse CD27 (CD27) BD Bioscience Cat #: 563605; 1:100
Anti-mouse CD8α (5H10) Pacific Orange Thermofisher Cat#: MCD0830 1:200
Anti-mouse CD4 (RM4-5) Qdot800 Thermofisher Cat#: Q22165 RRID:AB_2556521 1:250
Anti-mouse CD64 (X54-5/7.1) PECy7 BioLegend Cat#: 139314 1:100
Anti1:-human CD278 (ICOS) (C398.4A) BV510 Biolegend Cat #: 313525 1:50
Anti-Mouse CD11c (N418) BV711 Biolegend Cat#: 117349 1:100
Hamster Anti-Mouse CD183 (CXCR3-173) BV750 BD Biosciences Cat#: 747298 1:!00

	Anti-Mouse CX3CR1 (SA011F11) BV785 Biolegend Cat#: 149029 1:160<br>Rat Anti-mouse Siglec-F (E50-2440) BB515 BD Bioscience Cat#:564514 1:200<br>Anti-mouse TCRγδ (eBio-GL3) PECy5 Thermofisher Cat#: 15-5711-82 1:160<br>Anti-mouse CD301b/MGL2 (URA-1) PerCPCy5.5 BioLegend Cat#:146810 1:40<br>Anti-mouse CD273/PDL2 (B7-DC) PE BioLegend Cat#: 115565 1:20<br>Anti-mouse CD45R/B220 (RA3-6B2) APC/Fire810 BioLegend Cat#: 103278 1:200<br>Rat Anti-mouse CD62L (MEL-14) BUV563 BD Biosciences Cat#: 741230 1:200<br>Anti-mouse CD19 Antibody (6D5) Spark Blue 550 Biolegend Cat#: 115566 1:150<br>Mouse CXCR5 (614641) Alexa Fluor® 488 R&D Cat#: FAB6198G-100UG 1:100<br>Anti-mouse CD69 (H1.2F3) PE-Dazzle 594 Biolegend Cat#: 104535 1:100<br>Anti-mouse CD25 (PC61.5), PE-Cy5 Thermofisher Cat#: 15-0251-82 1:160<br>B7-2/CD86 (BU63) PE-Cy5.5 Novus Biological Cat#: NBP2-34569PECY55 1:100<br>Anti-mouse Tim-4 (RMT4-54) PECy7 Biolegend Cat#: 130010 1:50<br>Anti-mouse ST2 (DIH4) APC Biolegend Cat#: 146606 1:50<br>Anti-mouse CD206 (MMR) AF-647 Biolegend Cat#: 141712 1:100<br>Anti-mouse Ly-6C (HK1.4), PerCP Biolegend Cat#: 128028 1:!00<br>Anti-mouse Ly-6G (1A8), Spark NIR 685 Biolegend Cat#: 127666 1:200<br>Anti-mouse CD103, APC-R700 BD Biosciences Cat#: 565529 1:100<br>Anti-mouse KLRG1 (2F1/KLRG1), APC-Cyanine 7 Biolegend Cat#: 138436, 1:100
Validation	The validation information of all commercially antibodies used in this study are available on the provider websites.

Animals and other research organisms

Policy information about studies involving animals; ARRIVE guidelines recommended for reporting animal research, and Sex and Gender in Research

Laboratory animals	C57BL/6J, 129S1/SV1mJ and PWK/PhJ mice were purchased from The Jackson Laboratory (Bar Harbor, ME) and were housed under specific pathogen–free conditions with ad libitum access to food and water. All mouse lines were then bred onsite in a specific pathogen free (SPF) facility at National Institute of Health (NIH). The resulting littermates from the multiple breeding pairs were shipped to Princeton University where they were acclimated in a dedicated animal facility to temperatures and light cycles characteristics of summer in New Jersey (26℃ ± 1℃, and a 15-hour light/9-hour dark cycle). Following this, mice were randomly assigned to either remain in the institutional vivarium (lab mice) or released into the outdoor enclosures (rewilded mice).
Wild animals	No wild animals were used in the study.
Reporting on sex	For all rewilding experiments, only female mice were used to prevent unintended breeding in the rewilded environment. However,some immune phenotypes were repeated in laboratory conditions which included use of both male and female mice in the experiments.
Field-collected samples	No field collected samples were used in the study.
Ethics oversight	The protocols for mice breeding were approved by the NIAID Animal Care and Use Committee, Protocol Number, LPD 16E. The protocols for releasing the laboratory mice into the outdoor enclosure facility were approved by Princeton IACUC (protocol number 1982).

Note that full information on the approval of the study protocol must also be provided in the manuscript.

Plants

Seed stocks	N/A
Novel plant genotypes	N/A
Authentication	N/A

Flow Cytometry

Plots

Confirm that:

☒ The axis labels state the marker and fluorochrome used (e.g. CD4-FITC).

☒ The axis scales are clearly visible. Include numbers along axes only for bottom left plot of group (a 'group' is an analysis of identical markers).

☒ All plots are contour plots with outliers or pseudocolor plots.

☒ A numerical value for number of cells or percentage (with statistics) is provided.

Methodology

Sample preparation	For PBMC<br>Heparinized whole blood collected via the cheek bleeds was mixed with blood collected via cardiac puncture method. The combined blood samples were spun for 10 minutes at 1500 rpm and plasma was collected and stored at -80oC for further cytokine analysis. The cellular component re-suspended in PBS next underwent a density gradient separation process using the Lymphocyte Separation Media (LSMTM MP Biomedicals, LLC) according to manufacturer's instruction. Isolated PBMCs were washed twice in PBS and then used for downstream spectral cytometric analysis<br><br>For MLN<br>Single cell suspension from the mesenteric lymph nodes (MLNs) were prepared by mashing the tissues individually through a 70µm cell strainer and washing with RPMI. Cells were then washed with RPMI supplemented with 10% FCS.
Instrument	All samples were collected and acquired on an Aurora™ spectral cytometer (Cytek) 5-Laser Configuration
Software	OMIQ software and unsupervised clustering using Joe's Flow software - https://github.com/niaid/JoesFlow were used. PCA anlaysis for flow analysis were analyzed using R studio (version 2022.07.1). Code available in Github - https://github.com/oyeb2003/G-ERewilded-interaction-Project.
Cell population abundance	All samples were collected on an Aurora™ spectral cytometer (Cytek) and analyzed using the OMIQ software (https://www.omiq.ai/), data cleaning and scaling was done using algorithms like FlowCut within the OMIQ software. Traditional gating strategy was used to determine cellular proportions and they were then multiplied by cell counts which was enumerated using the Element HT5, Veterinary Hematology Analyzer (Heska) for determination of counts for the different cell types
Gating strategy	Gating/Clustering was done through and unsupervised clustering method (https://github.com/niaid/JoesFlow). Doublets (identified by plotting the height versus the area for forward and side scatter) and dead cells (by plotting fixable viability dye against the SSC-Height). CD45+ cell were identified and samples were sub-sampled for unsupervised clustering.<br><br>In situations where traditional gating was done, an example flow plot depicting gating strategy was provided in Extended Data Set 10. B cells were considered single, Live, CD45+, B220+; CD4 T cells were identified as Live, CD45+TCRb+CD4+; CD8 T cells as Live, CD45+TCRb+CD8+,

☒ Tick this box to confirm that a figure exemplifying the gating strategy is provided in the Supplementary Information.
